# The Effects of PEI Hollow Fiber Substrate Characteristics on PDMS/PEI Hollow Fiber Membranes for CO_2_/N_2_ Separation

**DOI:** 10.3390/membranes11010056

**Published:** 2021-01-14

**Authors:** Guoqiang Li, Wojciech Kujawski, Katarzyna Knozowska, Joanna Kujawa

**Affiliations:** 1 Faculty of Chemistry, Nicolaus Copernicus University in Toruń, 7, Gagarina Street, 87-100 Toruń, Poland; grantli@doktorant.umk.pl (G.L.); katkno@doktorant.umk.pl (K.K.); joanna.kujawa@umk.pl (J.K.); 2 National Research Nuclear University MEPhI, 31, Kashira Hwy, 115409 Moscow, Russia

**Keywords:** hollow fiber, PDMS coating, gas separation

## Abstract

The CO_2_ separation from flue gas based on membrane technology has drawn great attention in the last few decades. In this work, polyetherimide (PEI) hollow fibers were fabricated by using a dry-jet-wet spinning technique. Subsequently, the composite hollow fiber membranes were prepared by dip coating of polydimethylsiloxane (PDMS) selective layer on the outer surface of PEI hollow fibers. The hollow fibers spun from various spinning conditions were fully characterized. The influence of hollow fiber substrates on the CO_2_/N_2_ separation performance of PDMS/PEI composite membranes was estimated by gas permeance and ideal selectivity. The prepared composite membrane where the hollow fiber substrate was spun from 20 wt% of dope solution, 12 mL/min of bore fluid (water) flow rate exhibited the highest ideal selectivity equal to 21.3 with CO_2_ permeance of 59 GPU. It was found that the dope concentration, bore fluid flow rate and bore fluid composition affect the porous structure, surface morphology and dimension of hollow fibers. The bore fluid composition significantly influenced the gas permeance and ideal selectivity of the PDMS/PEI composite membrane. The prepared PDMS/PEI composite membranes possess comparable CO_2_/N_2_ separation performance to literature ones.

## 1. Introduction

With the rapid increase in the global population and the fast development of energy-intensive industries, the consumption of fossil fuels, i.e., coal, petroleum and natural gas, is drastically growing [1]. Consequently, the continuous increase in CO_2_ emissions is inevitable. As a result, according to the Intergovernmental Panel on Climate Change (IPCC) reports and the most comprehensive research, the CO_2_ concentration in the atmosphere is approaching 400 ppm which is higher than the safe level of CO_2_ concentration of 350 ppm [2]. Flue gas containing mainly CO_2_ and N_2_ from coal-fired power plants occupies 50–60% of the total global CO_2_ emission [3]. The excessive CO_2_ emission has caused anthropogenic climate change and global warming which has brought about various environmental problems, including rising sea levels, changes in ecosystems, loss of biodiversity and reduction in crop yields [4,5]. Therefore, there is an urgent need to reduce the CO_2_ emissions and the CO_2_ concentration in the atmosphere. Carbon capture and storage (CCS) is one of the most important technology used to reduce CO_2_ emissions [2,6]. In comparison to the traditional CO_2_ separation technologies e.g., absorption, adsorption, and cryogenic distillation, membrane technology possesses many advantages such as simple process operation, small footprint, energy efficiency and cost effectiveness [1,5,6,7].

Hollow fiber membranes have a promising future in various gas separation processes due to their advantages, e.g., high packing density and a self-supporting structure [8,9,10]. Hollow fibers are generally fabricated by using the dry-jet wet spinning technique. The structure, morphology, outer and inner diameters, wall thickness of hollow fibers were significantly influenced by the fabrication parameters, e.g., air gap length, bore fluid, dope composition and flow rates of dope and bore fluid [7,11,12,13,14]. Hasbullah et al. [12] found that the wall thickness of polyaniline (PAni) hollow fiber decreased while the skin layer thickness increased when the air gap length increased. These findings were attributed to the elongation stress resulting in a more packed structure and longer evaporation time during the dry phase separation in the air gap. Wang et al. [15] found that the addition of methanol, acetone, or a mixture of methanol/acetone into dope solution could tune the morphology of polyetherimide (PEI) hollow fibers resulting in enhanced gas separation performance. Kumbharkar et al. [7] found that increasing the solvent concentration in bore fluid is helpful for the formation of loose skin on the inner surface and the suppression of finger-like macro-voids in the substructure of polybenzimidazole (PBI) hollow fibers. Woo et al. [11] found that the addition of tetrahydrofuran (THF) is beneficial in order to suppress the finger-like macro-voids and to form a dense selective layer. The addition of LiCl salt is good for the suppression of finger-like macro-voids. In terms of the optimization of spinning conditions, the increase in bore flow rate results in an increase in the outer and inner diameter and a decrease in the wall thickness and dense layer thickness of a hollow fiber. The increasing dope flow rate led to larger outer and inner diameters and thicker fiber wall.

The development of membranes with high CO_2_ permeance is crucial to industrial applications. Therefore, the preparation of thin-film composite membranes consisting of a thin selective layer on a highly porous substrate, which provides mechanical strength, has attracted more and more attention in recent years [16,17]. To fabricate a defect-free thin film composite membrane, a highly permeable gutter layer can be introduced on the surface of the porous support, and a protective layer is needed to seal the pinholes on the selective layer [3,18,19]. A lot of research has been focused on the preparation of thin film composite hollow fiber membranes for gas separation [20,21,22,23,24,25,26].

Polydimethylsiloxane (PDMS) is a rubbery material with high gas permeability due to high flexibility of siloxane linkages and it is the most commonly used coating material for membrane gas separation processes due to its excellent adhesion property to the support. Moreover, PDMS possesses good thermal, chemical and oxidative stability [22,27,28]. Liang et al. [21] fabricated PDMS/PAN (polyacrylonitrile) thin film composite hollow fiber membranes by using the dip coating method. The prepared membranes were used for water vapor removal from humid air and gases. It was found that the composite membrane shows N_2_ permeance of about 280 GPU, O_2_/N_2_ selectivity of 2.2 and a water vapor permeance ranging from about 800 to 3700 GPU. Liang et al. [29] also prepared crosslinked PDMS/PAN thin film composite hollow fiber membranes for flue gas and air separations. The prepared composite membranes showed excellent O_2_ and CO_2_ permeances higher than 1000 and 5000 GPU, respectively, while the corresponding selectivities of O_2_/N_2_ and CO_2_/N_2_ are about 2 and 11, respectively. Roslan et al. [22] fabricated six different types of polysulfone (PSF) hollow fiber membranes from the same dope solution by varying the spinning parameters of air gap length, bore fluid flow rate, and collection speed to investigate the effect of hollow fiber substrate characteristics on the gas separation performance of thin film composite membranes. Subsequently, the prepared hollow fibers were dip coated with PDMS thin layer. It was found that DPMS coating significantly improved the selectivities of PSF hollow fiber membranes for CO_2_/CH_4_ and O_2_/N_2_ separation. PSF hollow fibers spun at a higher air gap (4 cm) and lower dope extrusion rate (1 mL/min) were found to be the best supports for PDMS coating owing to their good balance between gas permeance and gas selectivity. Chong et al. [30] fabricated PDMS/PSF composite hollow fiber membranes for oxygen enrichment. The prepared PDMS-coated membrane showed oxygen and nitrogen gas permeance of 18.31 and 4.01 GPU, respectively, with oxygen/nitrogen selectivity of 4.56. Li et al. [23] prepared PDMS/PAN hollow fiber composite membranes for the separations of CO_2_/N_2_ and O_2_/N_2_. The effects of prewetting agents, morphology and pore size distribution of substrate, and PDMS concentration and viscosity on the gas separation performance of the composite membranes were investigated. It was found that partial PDMS crosslinking and pre-wetting of PAN substrates with Fluorinert 72 (FC-72) or deionized water before dip coating can mitigate the solution intrusion in the dip coating process.

In this study, PEI hollow fibers were fabricated at various spinning conditions. The flow rate of bore fluid was especially chosen to investigate its effect on hollow fiber structure, since the flow rate of bore fluid was rarely investigated in the literature. PDMS/PEI composite hollow fiber membranes were prepared via the dip coating method. The structure, morphology, outer and inner diameters, wall thickness, and skin layer thickness of hollow fibers were characterized using various techniques to investigate the effect of substrate characteristics on the gas separation performance of the composite membrane.

## 2. Materials and Methods

### 2.1. Materials

Polyetherimide (PEI, Ultem 1000) pellets were kindly provided by Membrain s.r.o. (Stráž pod Ralskem, Czech Republic). N-methyl-2-pyrrolidone (NMP, 99.5%) was purchased from Linegal Chemicals Sp. z o.o. (Warsaw, Poland). Methanol and n-hexane were delivered by Alchem Grupa Sp. z o.o. (Toruń, Poland). Pure CO_2_ (99.999%) and N_2_ (99.999%) gases were purchased from Air Products Sp. z o.o. (Siewierz, Poland). The fast solidified epoxy resin Araldite 2000 and 3M EPX Quadro Mixing Nozzles were purchased from Farnell (Warsaw, Poland).

Elastosil LR 6240A (containing platinum catalyst) and Elastosil LR 6240B (containing crosslinker) were kindly provided by Wacker Chemie AG Polska Sp. z o.o. (Warsaw, Poland). According to the data provided by the producer, the viscosities of Elastosil LR 6240A and Elastosil LR 6240B are equal to 30–45 and 25–40 Pa s, respectively.

### 2.2. PEI Dope Solution Preparation

Dope solutions possessing various concentrations of PEI (16, 18, 20, 22, and 24 wt%) were prepared by dissolving PEI pellets in NMP solvent in a round bottom flask under refluxing conditions at 60 °C for 24 h. Prior to dissolving PEI pellets into NMP, PEI pellets were dried in the oven at 100 °C to remove residual moisture. The prepared dope solution was transferred into a laboratory screw cap bottle and left for 24 h for degassing.

### 2.3. PEI Hollow Fiber Preparation

**Figure 1:**
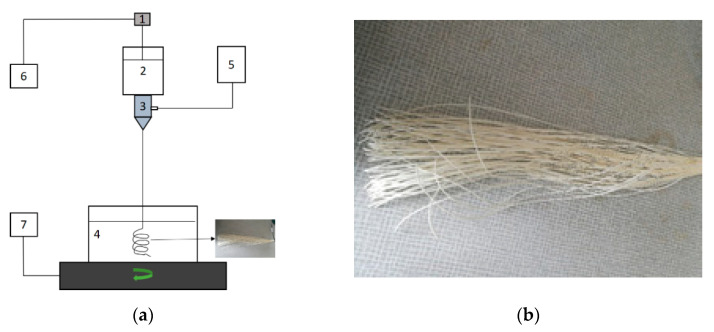
(**a**) Schematic representation of the home-built spinning system: (1) gear pump, (2) tank for spinning solution, (3) spinneret, (4) water coagulation bath, (5) syringe pump and tank for bore fluid, (6) control panel for a gear pump, (7) control panel for rotation of coagulation bath; (**b**) the prepared hollow fibers.

The PEI hollow fibers were prepared via the dry-jet wet spinning process by using a home-built spinning system (Figure 1). Polymer concentration, bore fluid composition, and flow rate of bore fluid were chosen as variants and investigated in this study. The names of the fabricated hollow fibers and their corresponding values of variants are gathered in Table 1. The spinning conditions are shown in Table 2. In the spinning process, a gear pump was used to deliver the dope solution at a specific extrusion rate from the stainless steel reservoir to a spinneret. The bore fluid was delivered into the spinneret simultaneously by using a syringe pump. The as-spun hollow fibers went through an air gap and fell free into a coagulation bath containing distilled water at room temperature. The prepared hollow fibers were cut and soaked in another water bath for 2 days to remove the remaining NMP solvent. The solvent exchange was applied as a post-treatment on hollow fibers to avoid the collapse of the hollow fiber structure during the drying process. The hollow fibers were taken out from the water bath and immediately immersed in methanol for 12 h. Then the methanol-wet hollow fibers were immersed in hexane for another 12 h. Finally, hollow fibers were taken from hexane and dried at room temperature before further investigations.

### 2.4. Fabrication of PDMS/PEI Composite Hollow Fiber Membranes

Elastosil LR 6240 A and Elastosil LR 6240 B in the mass ratio 1:10 were dissolved in hexane to prepare 15 wt% PDMS solution. The solution was prepared by stirring for 2 h at room temperature. The PDMS/PEI composite hollow fiber membranes were fabricated using a dip-coating method. First of all, a 30 cm long PEI hollow fiber was prepared, and one end of the hollow fiber was sealed with epoxy resin. After the solidification of epoxy resin, the other end of the hollow fiber was attached to a metal holder. Then the single PEI hollow fiber was vertically immersed into the PDMS solution for 10 min at room temperature. Finally, the PDMS coated hollow fiber was slowly taken from the coating solution and dried in air for at least 48 h to remove the solvent and fully cure the PDMS.

### 2.5. Characterization of PEI Hollow Fibers and PDMS/PEI Composite Membranes

The morphology of the fabricated PEI hollow fibers and PDMS/PEI composite membranes were analyzed by using Scanning Electron Microscope (SEM)—LEO 1430 VP microscope (Leo Electron Microscopy Ltd., Cambridge, UK). The scanning was performed at an accelerating voltage of 30 keV. To analyze the cross-section of hollow fiber, the sample was prepared by fracturing the hollow fiber in liquid nitrogen. Prior to the analysis, the sample was sputtered with a conductive layer (thickness in the range of 2–6 nm) of Au/Pd (80/20 composition). The inner diameter, outer diameter, wall thickness, outer skin layer thickness, and inner skin layer thickness of hollow fiber were measured from SEM pictures by using ImageJ software. The thicknesses of PDMS layers at the top part and bottom part were also measured by using ImageJ software.

The contact angle (CA) of the inner and outer surfaces of hollow fibers were measured by using a Theta Flex Tensiometer (Biolin Scientific, Gothenburg, Sweden) at room temperature. The sample was prepared by opening the hollow fiber by using a scalpel and mini scissors. Attension Theta (OneAttension Version 4.02, Gothenburg, Sweden) software was used for data acquisition and processing. Water with surface tension equal to 72.5 mN m^−1^ was selected as testing liquid.

### 2.6. Module Fabrication and Gas Permeance Measurements

**Figure 2:**
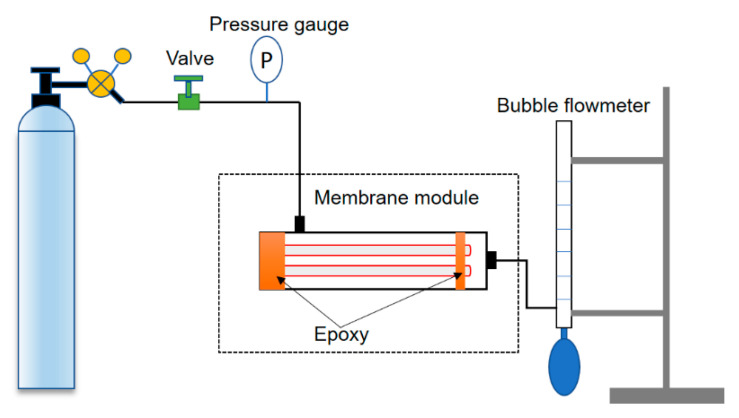
The scheme of laboratory set-up for gas permeance measurements of hollow fiber membranes.

The modules were fabricated according to the following procedure. A total of 2 hollow fibers with a length of 15–20 cm were assembled as a bundle. One hollow fiber bundle was placed in a glass tube. Both ends of the glass tube were sealed with a 5 min fast solidified epoxy resin (Araldite, Winterthur, Switzerland). Then one end of the glass tube was opened by using a scalpel before the complete solidification of epoxy resin. The prepared module was fitted into a home-made apparatus as shown in Figure 2 for gas permeance measurements. Pure N_2_ and CO_2_ were used for the single gas permeance tests. The trans-membrane pressure was set as 2 bar for all measurements at room temperature 25 °C. To ensure the accuracy of experiments, the gas permeance measurements were conducted 3 times in the stabilized condition. The permeances, P/d, of gases through the hollow fiber module were determined using a bubble flow meter and calculated using Equation (1):

(1) $$\frac{P}{d}=\frac{Q}{\Delta \mathrm{pA}}=\frac{Q}{2n\pi \mathrm{rl}\Delta p}$$

where P is the permeability (Barrer); d is the thickness of membrane selective layer (cm); Q is the flux of gas permeation rate (cm^3^ (STP)/s); Δp is the pressure difference across the membrane (cmHg); A is the effective membrane area (cm^2^); n is the number of hollow fiber; r is the outer radius (cm) of hollow fiber; P/d is the gas permeance expressed in GPU (1 GPU = 10^−6^ cm^3^ (STP) cm^−2^ s^−1^ cmHg^−1^).

The ideal selectivity α is defined as the permeability coefficient or permeance ratio of two pure gases (Equation (2))

(2) $$\alpha_{12}\;=\;\frac{{\left(P/d\right)}_{1}}{{\left(P/d\right)}_{2}}=\frac{P_{1}}{P_{2}}$$

## 3. Results and Discussion

### 3.1. PEI Hollow Fiber Substrate

#### 3.1.1. The Effect of Polymer Solution Concentration

**Figure 3:**
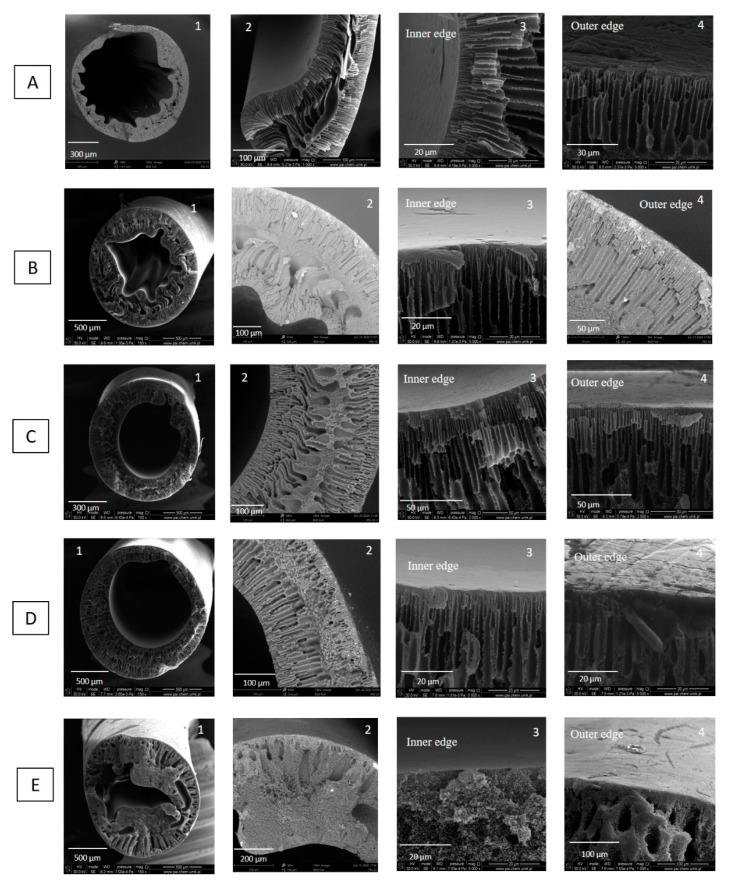
SEM micrographs of cross section of PEI hollow fibers spun from various concentrations of polymer solution—(**A**) HF1—16 wt%, (**B**) HF2—18 wt%, (**C**) HF3-2—20 wt%, (**D**) HF4—22 wt%, (**E**) HF5—24 wt% (bore fluid—water and bore fluid flow rate 6 mL/min).

The thermodynamic and kinetic principles involved in phase inversion technique such as polymer–solvent interactions, solvent–coagulant interactions, and the concentration and viscosity of dope affect membrane morphology [31,32]. To investigate the influence of concentration of the polymer solution on the hollow fiber formation, hollow fibers were spun from various concentrations of the polymer solution. The rest of the spinning conditions were kept constant (Table 2). Figure 3 shows the cross-section morphology as a function of PEI solution concentration, i.e., 16 wt%, 18 wt%, 20 wt%, 22 wt%, and 24 wt%. The following conclusions can be drawn. Hollow fibers spun from 16 wt%, 18 wt%, and 20 wt% of PEI solution possess similar morphology, i.e., finger-like macrovoids in the bulk of the hollow fibers underneath the inner and outer skin layers, and tear-like macrovoids in the middle part of the hollow fiber wall. However, when the polymer concentration increased from 16 wt% to 20wt%, the tear-like macrovoids became smaller, and the finger-like macrovoids became shorter (Figure 3A2–C2). This is because the water intrusion is suppressed to some extent, resulting from the greater viscoelasticity of a more concentrated polymer solution [33]. Especially, in the case of hollow fibers spun from 20 wt% of PEI solution, a symmetric structure appeared in the hollow fiber wall (Figure 3C2). The symmetric structure is beneficial to the mechanical stability of hollow fibers which is used as a support layer. Jamil et al. [34] found that NMP has weaker interaction towards PEI, hence, it formed instant demixing and migrated to water coagulant, which created finger-like pores. When the polymer solution concentration increased to 22 wt%, the part with tear-like macrovoids was replaced by a part with sponge-like microporous structure and the finger-like macrovoids near lumen side were longer than the ones near shell sides. The decrease in the length of finger-like macrovoids near the shell side is attributed to the fast formation of relatively dense layer when hollow fibers were passing through the air gap, which impeded the intrusion of nonsolvent (water) into hollow fibers. Hollow fibers spun from 24 wt% PEI solution possess the sponge-like porous structure with a small number of tear-like macrovoids near lumen and shell sides. The sponge-like pores were formed due to the slow exchange of solvent at higher chain orientations [31]. In the hollow fiber fabrication process, the increase in polymer solution can reduce the number and size of macrovoids. Consequently, the macrovoids can be eliminated by increasing the polymer solution concentration. As it is shown in Figure S1, hollow fibers HF1, HF2, HF3-2, HF4, and HF5 possessed a skin layer on the inner and outer surfaces. The formation of skin layers on both the inner and outer side of hollow fibers resulted from the fast precipitation process. Water was used as an inner and outer coagulant in the spinning process. It is a strong nonsolvent that induced the strong kinetics and thermodynamic effects between polymer–solvent and non–solvent (water) during the phase inversion process, which accelerated the precipitation rate the polymer solution [35].

Naim et al. [35] and Bakeri et al. [36] investigated the effect of polymer concentration on the structure and performance of PEI hollow fiber membranes. Bakeri et al. [36] found that the phase separation took place in an earlier stage of solvent–nonsolvent exchange for a higher polymer concentration. However, the higher viscosity of the solution delays the solvent–polymer demixing by slowing down the solvent–nonsolvent exchange process. The thickness and density of the skin layer increased with the increase in polymer concentration. All prepared membranes possessed finger-like macrovoids, which extended from the inner and outer surfaces to the middle of the hollow fiber wall [36]. Naim et al. [35] found that the fine line sponge-like structure was formed in the middle intersection of the finger-like arrangement and varied in terms of the thickness when the polymer concentration increased. Their observation is similar to ours (Figure 3). The finger-like structure of the hollow fibers resulted from the rapid phase inversion process due to the low viscosity of the respective polymer solution. Moreover, water was used as the internal and external coagulants in the spinning process, the strong non-solvent has accelerated the phase inversion rate. The low viscosity of the polymer solution (13–16 wt%) to some extend contributed to the similarity of the finger-like structure. It is believed that the thermodynamic and kinetics effects played crucial roles in determining the membrane structure which can be manipulated based on the parameters applied in the spinning process, e.g., coagulation medium, air gap, bore fluid composition, spinneret size, and fibers collection methods (spin drum or free falling) [35].

**Figure 4:**
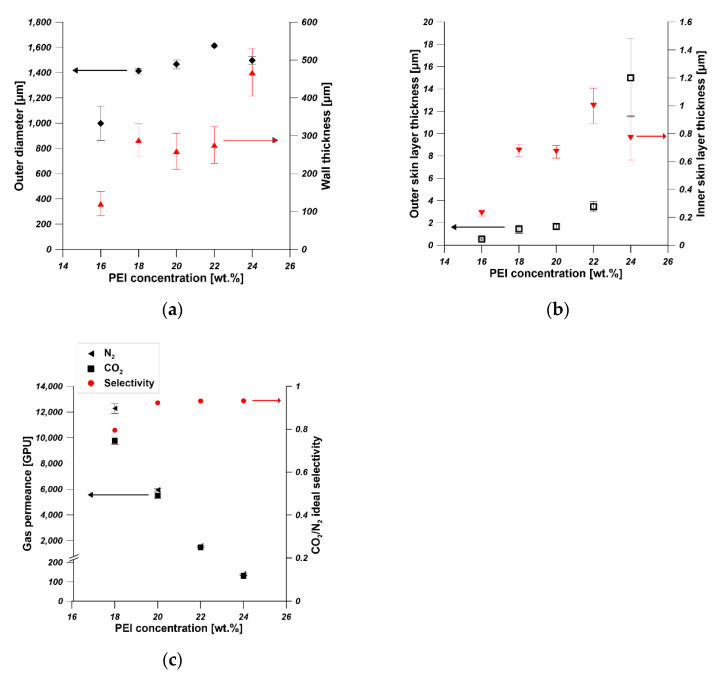
The effect of PEI concentration in dope solution on (**a**) the outer diameter and wall thickness, (**b**) the skin layer thickness (**c**) the gas permeance and ideal selectivity of hollow fibers (bore fluid—water and bore fluid flow rate 6 mL/min).

The dimension parameters of hollow fibers i.e., outer diameters, wall thickness, outer skin layer thickness and inner skin layer thickness, were measured by using ImageJ software. As is shown in Figure 4a, the outer diameter increased with the increase in PEI concentration. The outer diameter was in the range of 1400–1600 µm when the PEI concentration was in the range of 18–24 wt%. The influence of dope concentration on the outer diameter of hollow fibers weakened when the dope concentration is higher. The wall thickness of hollow fiber increased from 400 µm to 800 µm when the PEI concentration increased from 16 wt% to 18 wt%. Then it was stabilized at around 800 µm even if the PEI concentration increased from 18 wt% to 22 wt%. HF5 possessed the highest wall thickness equal to 476 µm. The effect of PEI concentration from 16 to 22 wt% on the outer diameter and wall thickness was weakened at higher PEI concentration, which can be explained in terms of viscosity of dope and the phase separation process. The viscosity of the PEI solution increased when the PEI concentration increased [36]. The phase separation took place at an earlier stage of solvent–nonsolvent exchange for a higher polymer concentration [35]. Therefore, the increased viscosity and the faster phase separation restrict the changes of outer diameter and the wall thickness of hollow fibers in the spinning process.

The thicknesses of outer and inner skin layers increased from 0.55 µm to 15 µm and from 0.2 µm to 1 µm, respectively, when the PEI concentration increased from 16 wt% to 24 wt% (Figure 4b). The increase in the skin layers can be attributed to the increased viscosity and slower solvent–nonsolvent exchange rate [36]. The gas permeance test reflecting the skin layer properties showed that the permeances of N_2_ and CO_2_ decreased with the increase in PEI concentration, which is in accordance with the skin layer thickness observed from SEM pictures (Figure 4c). The gas permeance is also influenced by the pore structure of hollow fibers. The finger-like pores promote the gas permeance [31], which is confirmed in Figure 3. The sponge-like hollow fiber showed the lowest N_2_ and CO_2_ permeance between 100 GPU and 200 GPU. The prepared hollow fibers showed CO_2_/N_2_ selectivity lower than one and high N_2_ and CO_2_ permeance except for HF5, which reflected the high porosity of the prepared hollow fibers (Figure 3). During the gas permeance test, the feed gas went from the shell side to the lumen side. The gas permeance of HF1 was too high to be measured, and the HF1 collapsed at 2 bar during the gas permeance test. Therefore, hollow fibers fabricated from 16 wt% of PEI solution are not suitable as mechanical supports of composite membranes.

As was discussed before, hollow fibers fabricated from 20 wt% of PEI solution showed desirable pore structure (Figure 3C) and relatively high gas permeance around 6000 GPU. Therefore, 20 wt% of PEI solution was chosen as the optimal dope for hollow fiber preparation, and further investigation were conducted on hollow fibers fabricated from 20 wt% of PEI solution.

#### 3.1.2. The Effect of Bore Fluid Flow Rate

**Figure 5:**
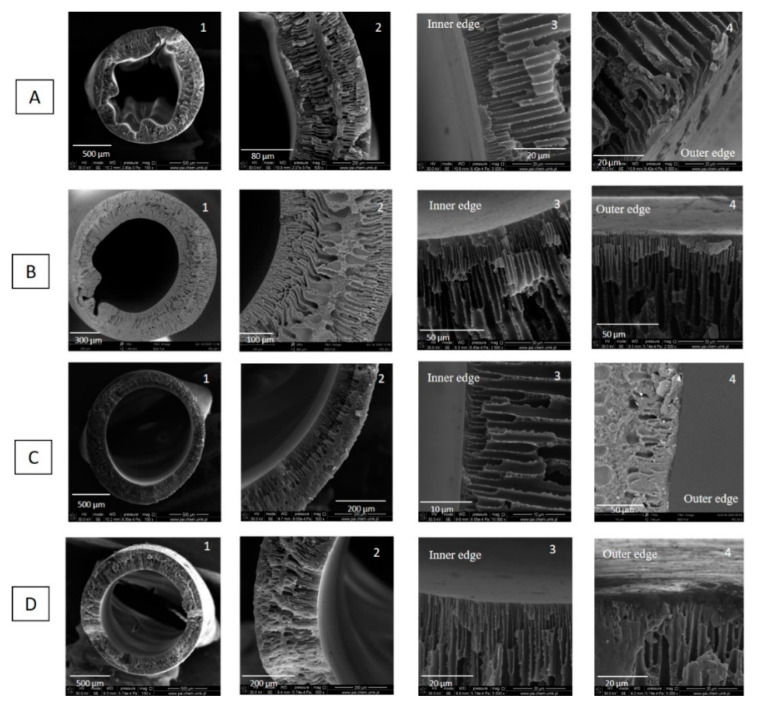
SEM micrographs of cross section of PEI hollow fibers spun at various flow rates of bore fluid. (**A**) HF3-1—3 cm^3^/min, (**B**) HF3-2—6 cm^3^/min, (**C**) HF3-3—9 cm^3^/min, (**D**) HF3-4—12 cm^3^/min (PEI concentration—20 wt% and bore fluid—water).

To investigate the influence of bore fluid flow rate on the hollow fiber formation, hollow fibers were spun from 20 wt% PEI solution and at various bore fluid flow rates. The rest of the spinning conditions were kept constant (Table 2). Figure 5 shows the cross-section morphology as a function of bore fluid flow rate, i.e., 3 cm^3^/min, 6 cm^3^/min, 9 cm^3^/min, and 12 cm^3^/min. It was observed that when the bore fluid flow rate increased, the inner contour of the cross-section became circular in shape (Figure 5C1,D1) from a corrugated one (Figure 5A1,B1) and the wall of hollow fiber became thinner and homogeneous. Bonyadi et al. [37] investigated the corrugation phenomenon in the inner contour of hollow fibers during the non-solvent induced phase separation process. They proposed two possible instability mechanisms to elucidate the deformations of the inner contour of hollow fibers in the spinning process. According to their theory, the instability arose from the elastic, hydrodynamic, mass transfer, and solidification processes. The effects of air-gap distance, bore fluid composition, external coagulant, take-up speed, and dope concentration on the formation of corrugation in the inner contour were explained in detail based on the proposed theory [37]. Their theory could be used to explain the effect of bore fluid flow rate on the corrugation phenomenon in the inner contour in the spinning process. When the bore fluid flow rate was small, the mass transfer between the dope and bore fluid is slow. The bore fluid penetration into the polymer solution was not homogeneous. The polymer solution matrix was divided into regions which have different penetration and contact area with bore fluid. In the region possessing deeper penetration and increased contact area with bore fluid, the solvent–nonsolvent exchange rate between dope and bore fluid is faster and the pressure induced by precipitation in these regions is higher resulting in the deformation of the inner contour [37]. With the increase in bore fluid flow rate, the mass transfer was enhanced, and the solvent–nonsolvent exchange rate between dope and bore fluid became higher and more homogeneous. Therefore, the pressure-induced by precipitation was similar, inhibiting the deformation in the inner contour. On the other hand, the increased solvent–nonsolvent exchange rate resulted in a more rigid elastic cylindrical shell in the inner part of the dope and a more viscous region in the middle part of the dope. Consequently, the initial instabilities were inhibited, and the effect of radial inward shrinkage force generated in the outer coagulant on the deformation of hollow fiber was weakened [37].

Hollow fibers (HF3-1, HF3-2, HF3-3, and HF3-4) possessed finger-like macrovoids near lumen and shell sides. Wang et al. [38] also observed a typical bulk structure of double rows of finger-like macrovoids with a compact skin at both the outer and inner surface of polyimides hollow fibers spun with water as bore fluid. This is because water is a strong nonsolvent. Both the inner and the external interfaces of the dope undergo instantaneous phase separation, which forms a thin and dense layer on both surfaces of hollow fiber with a finger-like structure of the sublayer. The finger-like macrovoids near the shell side are shorter in comparison with the ones near the lumen side. This is due to the formation of dense outer layer in the air gap impeding the water intrusion and the different contact times between the dope and coagulant (water) since the outer surface of the dope went through an air gap during the spinning process [38]. With an increase in bore fluid flow rate, the finger-like macrovoids near the lumen became longer, which is attributed to the stronger nonsolvent (water) intrusion and the facilitated mass transfer resulting from higher bore fluid flow rate [39]. The hollow fibers spun at a lower bore fluid flow rate (3 cm^3^/min) possess thicker layers with a microporous structure in the middle part of the hollow fiber wall. The microporous structure in the middle part of hollow fiber wall became thinner with the increase in bore fluid flow rate. The number and size of tear-like macrovoids significantly decreased when the bore fluid flow rate was at 9 and 12 cm^3^/min. The increase in flow rate can influence the cross-section profile and the size and shape of macrovoids.

Figure S2 reveals the morphology of inner and outer surfaces of HF3-1, HF3-2, HF3-3, and HF3-4. All hollow fibers possessed outer and inner skin layers since water as a strong nonsolvent was used as the bore fluid. The increase in bore fluid flow rate did not affect the surface morphology of hollow fibers significantly.

**Figure 6:**
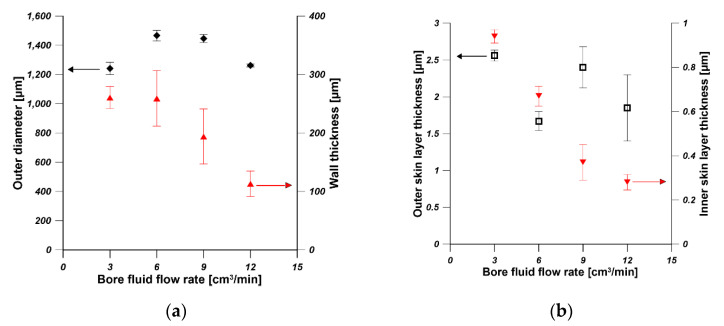

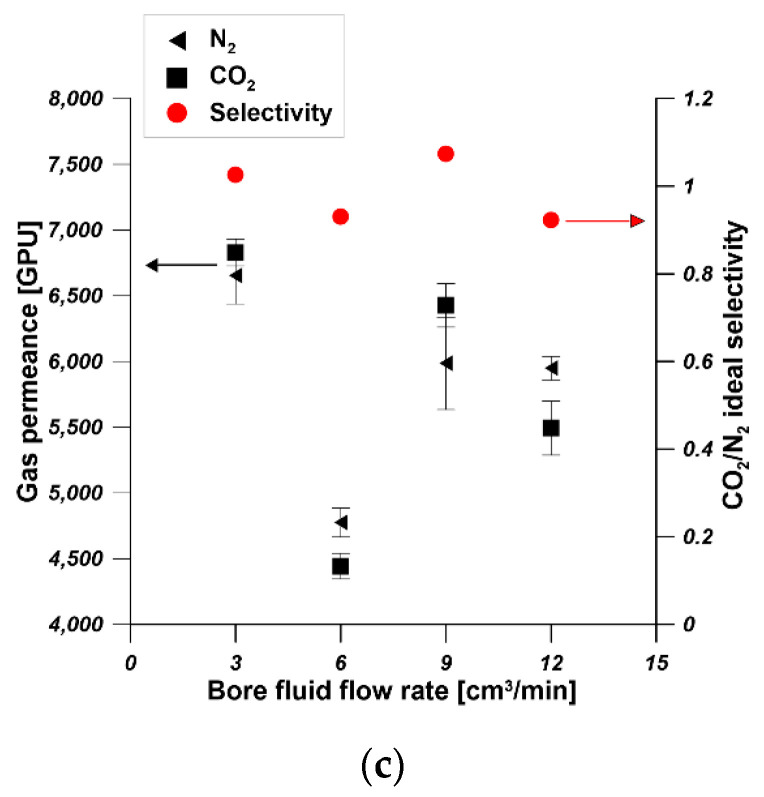
The effect of bore fluid flow rate on (**a**) the outer diameter and wall thickness, (**b**) the skin layer thickness (**c**) the gas permeance and ideal selectivity of hollow fibers (PEI concentration—20 wt% and bore fluid—water).

Figure 6a shows the effect of bore fluid flow rate on the outer diameter and wall thickness of hollow fibers spun from 20 wt% using water as bore fluid. With the increase in bore fluid flow rate, the outer diameter was slightly influenced, however, the wall thickness decreased significantly. Consequently, the inner diameter of hollow fibers increased with the increase in bore fluid flow rate. Wang et al. [39] observed the increased inner diameter and the reduced wall thickness of the as-spun polybenzimidazole (PBI) hollow fibers. Bildyukevich et al. [40] also found that the capillary diameter increased and wall thickness decreased with an increase in the rate of bore fluid at a constant polysulfone solution feed rate. This is due to the fact that the solidification rate at the inner surface increases with the increasing bore fluid flow rate since the mass transfer is facilitated. Therefore, an increase in bore fluid flow rate results in the increase in the inner diameter with a reduced wall thickness and slightly stretched outer skin [39]. As Figure 6b shows, the thickness of the outer skin layer was in the range of 1.5–2.5 µm, and the thickness of the inner skin layer decreased from 0.94 µm to 0.28 µm. The slight decrease in inner skin layer is due to the increased solvent–nonsolvent exchange rate resulting from the facilitated mass transfer. Figure 6c shows that hollow fibers spun from 20 wt% using various bore fluid flow rates possessed very high gas (N_2_ and CO_2_) permeance in the range of 4500–7000 GPU with no selectivity.

**Figure 7:**
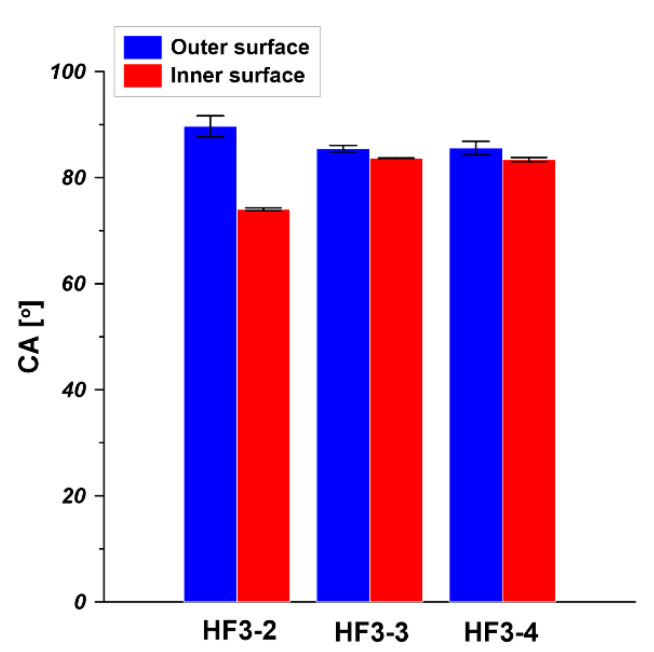
Water contact angle (CA) of PEI hollow fibers spun at various bore fluid flow rates (HF3-2—6 mL/min, HF3-3—9 mL/min, HF3-4—12 mL/min, PEI concentration—20 wt% and bore fluid—water).

The water contact angle (CA) of the outer surface and inner surface of hollow fibers i.e., HF3-2, HF3-3, and HF3-4, was measured at room temperature. HF3-2, HF3-3, and HF3-4 were spun from 20 wt% PEI solution at a bore fluid flow rate equal to 6 mL/min, 9 mL/min, and 12 mL/min, respectively. As is shown in Figure 7, the CA of the outer surface is between 85° and 89°. Considering the measurement deviation, it can be concluded that the increase in bore fluid flow rate barely affects the outer surface CA since all hollow fibers went through the same air gap then went into the water coagulant bath. The inner surface CA slightly increased from 74° to 84° when the bore fluid flow rate increased from 6 mL/min to 9 mL/min and 12 mL/min. This is because corrugation exists in the inner surface (seen from cross-section in Figure 5), resulting in slightly different morphology and surface roughness compared to the inner surface of the hollow fiber spun at a higher bore fluid flow rate. It is reported that the surface morphology and roughness affect the contact angle value [41]. The CA of the outer surface and the inner surface is practically the same due to the absence of corrugation on the inner surface (seen from the cross-section in Figure 5) and the use of water as inner and outer coagulant. Similar results were obtained in other research [42,43,44]. Qtaishat et al. [42] prepared PEI flat sheet membrane from 12 wt% of PEI solution. It was found that the CA for the top layer and the bottom layer was 80.04° ± 4.55° and 72.83° ± 2.62°, respectively. Bakeri et al. [43] fabricated PEI hollow fiber membranes from 14.5 wt% PEI solution. They found that the inner surface CA of PEI hollow fiber was 80.6° ± 2.5°.

#### 3.1.3. The Effect of Bore Fluid Composition

**Figure 8:**
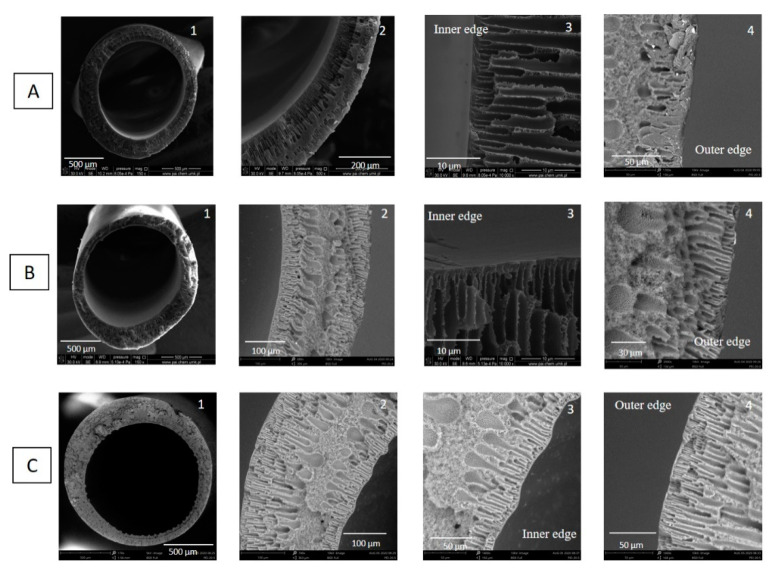
SEM micrographs of cross section of PEI hollow fibers spun with various compositions of bore fluid. (**A**) HF3-3—H_2_O, (**B**) HF3-5—H_2_O/N-methyl-2-pyrrolidone (NMP) 50/50wt%, (**C**) HF3-6—H_2_O/NMP 30/70wt% (PEI concentration—20 wt% and bore fluid flow rate—9 mL/min).

To investigate the influence of bore fluid composition on the lumen side and the region near the lumen side, hollow fibers were spun from 20 wt% PEI solution using different bore fluids. The rest of the spinning conditions were kept constant (Table 2). Figure 8 shows the cross-section morphology as a function of bore fluid composition, i.e., H_2_O, H_2_O/NMP 50/50 wt%, H_2_O/NMP 30/70 wt%. All hollow fibers possessed finger-like macrovoids near lumen and shell sides, tear-like macrovoids underneath the finger-like macrovoids, and a microporous structure in the middle part of the hollow fiber wall. When the water fraction in bore fluid decreased from 100 wt% to 30 wt%, the finger-like macrovoids near the lumen side became shorter due to the weakened nonsolvent (water) intrusion. The size of tear-like macrovoids increased, and the thickness of the microporous structure in the middle part of hollow fiber walls increased. With the addition of NMP into the bore fluid, the coagulant effect of bore fluid became weaker, resulting in delayed phase separation in the inner region of the dope. What is more, the addition of NMP into the bore fluid inhibited the mass transfer between the inner coagulant and the polymer solutions because the driving force to water inflow and solvent outflow decreased [38,45].

Bore fluid directly contacts with the inner region of dope in the spinning process and significantly affects the morphology of the inner surface of hollow fibers. Therefore, the morphology of the inner surface and the inner structure of hollow fibers can be influenced by controlling the composition of bore fluid [38]. Figure S3 reveals the inner and outer surface morphologies of hollow fibers spun with various bore fluids, e.g., H_2_O, H_2_O/NMP 50/50 wt%, H_2_O/NMP 30/70 wt%. The morphologies of outer surfaces of hollow fibers are similar since the as-spun hollow fibers went through the same air gap and into the external coagulant (water) bath. The instantaneous phase separation occurred in this process, and a dense skin layer was formed on the outer surface of hollow fibers. The influence of bore fluid composition on the inner surface morphology can be observed from the SEM pictures. The morphologies of the inner surfaces were slightly different, and pores started appearing with the addition of NMP into bore fluid. Similar results were obtained by Yong et al. [46]. They found that the inner surface porosity increased with the increase in solvent (NMP) concentration in bore fluid. The addition of solvent into bore fluid weakened the coagulant effect and resulted in the delayed phase separation in the inner region of the dope [46].

**Figure 9:**
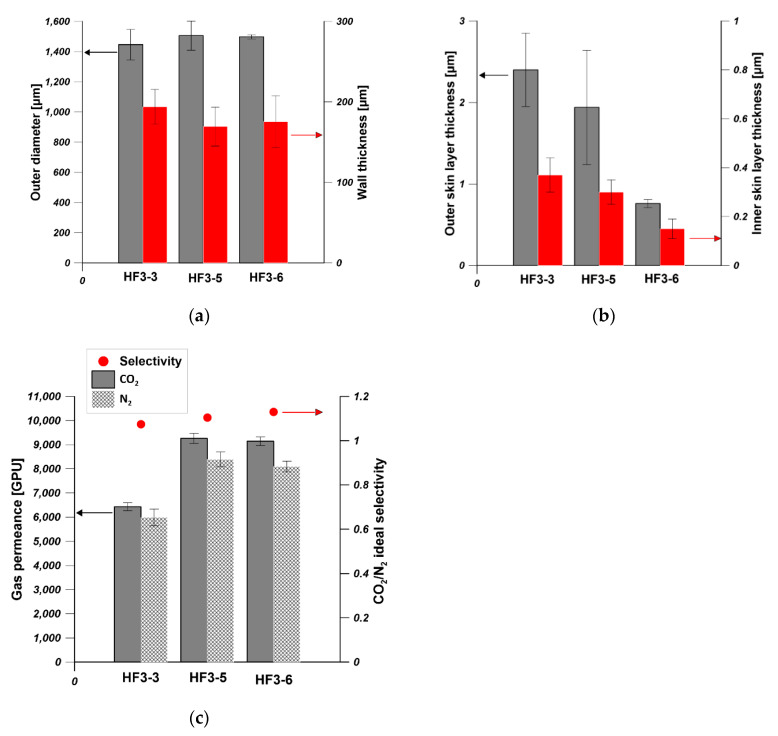
The effect of bore fluid composition on (**a**) the out diameter and wall thickness, (**b**) the skin layer thickness (**c**) the gas permeance and ideal selectivity of hollow fibers (HF3-3—H_2_O, HF3-5—H_2_O/NMP—50/50 wt%, and HF3-6—H_2_O/NMP—30/70 wt%, PEI concentration—20 wt% and bore fluid flow rate—9 mL/min).

As Figure 9a shows, the addition of NMP into bore fluid did not affect the outer diameter and wall thickness of hollow fibers. The slight decrease in the inner skin layer thickness resulted from the delayed phase separation process in the inner region of dope. Even the bore fluid did not contact the outer surface of hollow fiber directly, the thickness of the outer skin layer slightly decreased (Figure 9b). It can be seen from Figure 9c that the addition of NMP into bore fluid increased the CO_2_ and N_2_ permeance from 6500 GPU and 6000 GPU to 9000 GPU and 8000 GPU, respectively. The slight increase in gas permeance might result from the increased porosity on the inner surface of hollow fibers (Figure S3) and the slight decrease in the skin layer on the inner and outer surfaces of hollow fibers (Figure 9b). Similar results were found by Yong et al. [46]. They observed that the fiber spun with a higher solvent (NMP) in the bore fluid possessed a higher O_2_ and CO_2_ permeance. This phenomenon is directly related to the highly porous inner surface structure as a consequence of the delay demixing [46]. All hollow fibers possessed very high gas permeance over 6000 GPU, indicating the lower resistance for gas transport.

**Figure 10:**
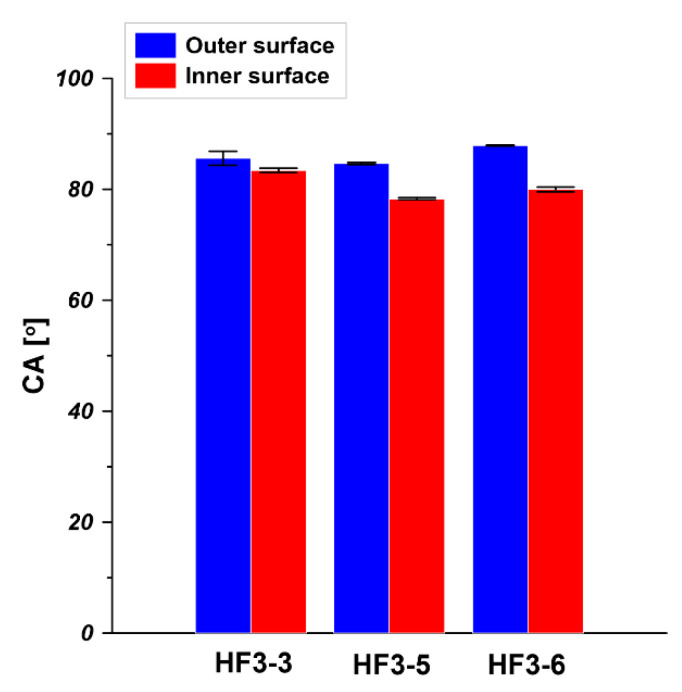
Water contact angle (CA) of PEI hollow fibers spun by using H_2_O, H_2_O/NMP—50/50 wt%, and H_2_O/NMP—30/70 wt% as bore fluids for HF3-3, HF3-5, and HF3-6, respectively (PEI concentration—20 wt% and bore fluid flow rate—9 mL/min).

As is shown in Figure 10, the CA of the outer surface is around 86°. The addition of NMP into bore fluid did not influence the CA outer surface since the outer surface was formed at the same spinning condition. However, the addition of NMP into bore fluid should affect the inner surface properties since the inner surface was in direct contact with bore fluid. It was observed that when the NMP content in bore fluid increased from 0 wt% to 50 wt% and 70 wt%, respectively, the CA of the inner surface decreased from 83° to 78° and 80°, respectively. The influence of NMP addition into bore fluid on the CA of the inner surface was due to the change of surface morphology which resulted from the delayed phase separation in the inner region of the dope [46].

### 3.2. PDMS/PEI Composite Hollow Fiber Membranes

To investigate the effect of hollow fiber substrate characteristics on the gas separation performance of PDMS/PEI composite hollow fiber membranes, a PDMS layer was dip coated on the outer surface of various hollow fibers which were fabricated at different spinning conditions. The designations of PDMS/PEI composite membranes together with the corresponding hollow fiber substrates are gathered in Table 3.

#### 3.2.1. Morphology

**Figure 11:**
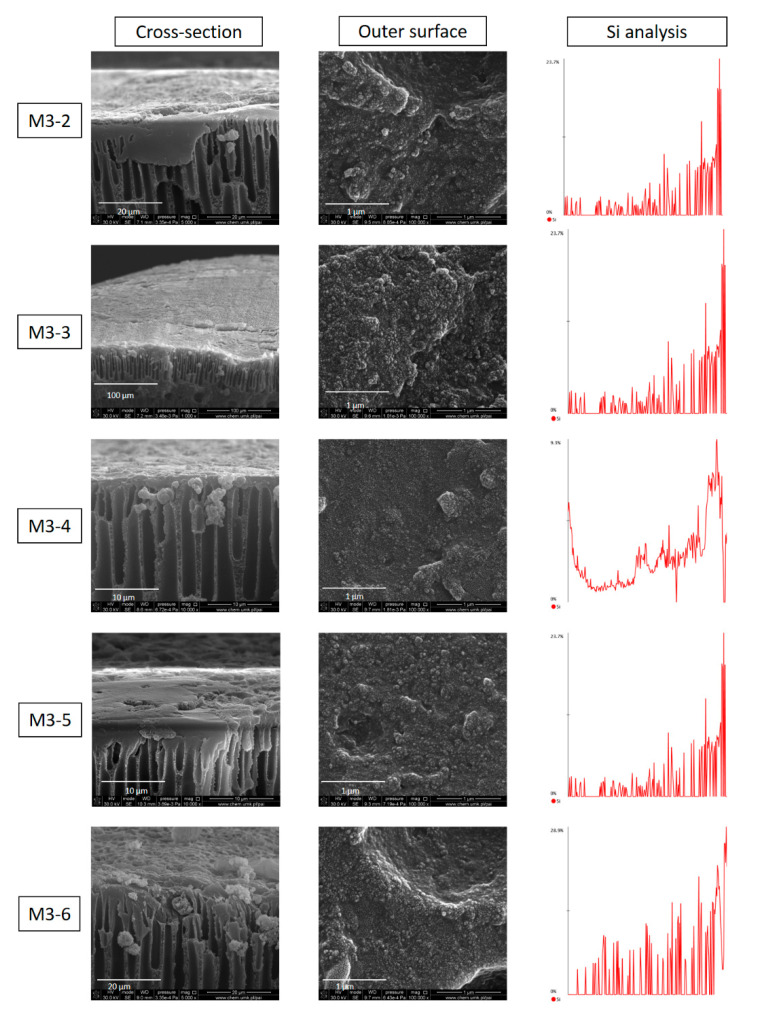
The morphology of cross-section and outer surface of a PDMS/PEI composite membrane and the Si distribution made by using a line scan from the inner surface to the outer surface (PDMS concentration—15 wt% and coating time—10 min).

The morphology of the PDMS/PEI composite membrane was characterized by using SEM and the Si element identification was performed from the inner surface to the outer surface of the composite membrane by using the line scan mode of EDX (Energy-dispersive X-ray spectroscopy). As it is shown in Figure 11, the PDMS selective layer was successfully coated on the outer surface of hollow fiber support, which is observed from the cross-section and confirmed from the gas separation performance (Figure 14). The element identification confirmed that the PDMS layer was formed on the outer surface of hollow fibers due to the existence of abundant Si elements on the outer surface (Figure 11). Moreover, the Si distribution curve indicates the occurrence of PDMS solution intrusion into hollow fibers in the dip-coating process. The intrusion of PDMS solution into porous support was also studied by Vankelecom et al. [47].

**Figure 12:**
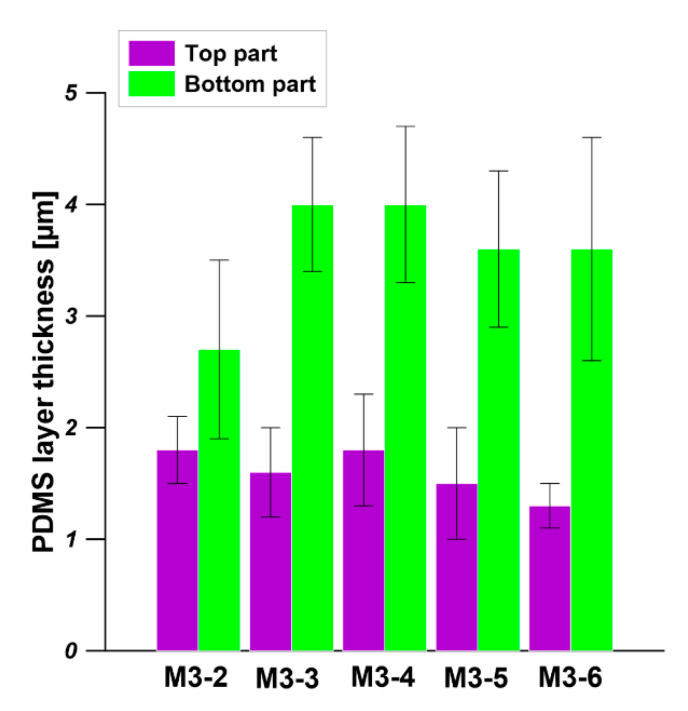
The thickness of the PDMS layer measured from the top part and bottom part of the PDMS/PEI composite hollow fiber membrane. The dip coating conditions: PDMS solution concentration—15 wt% and coating time—10 min.

As is shown in Figure 12, the PDMS layer thickness of the top part and bottom part of PDMS/PEI composite hollow fiber membrane is in the range of 1.5–2 µm and 3–4 µm, respectively. The difference in the PDMS layer thickness arose from the dip coating process. In the dip coating process, the bottom part was always close to the solution reservoir while the top part was relatively far from the coating solution. The thickness of the PDMS layer is related to the position of the drying line. The interplay of several parameters e.g., viscous force, solvent evaporation and draining, surface tension, gravity and hydrodynamic factors in the layer deposition region, governs the layer thickness and the position of the drying line [48,49,50].

**Figure 13:**
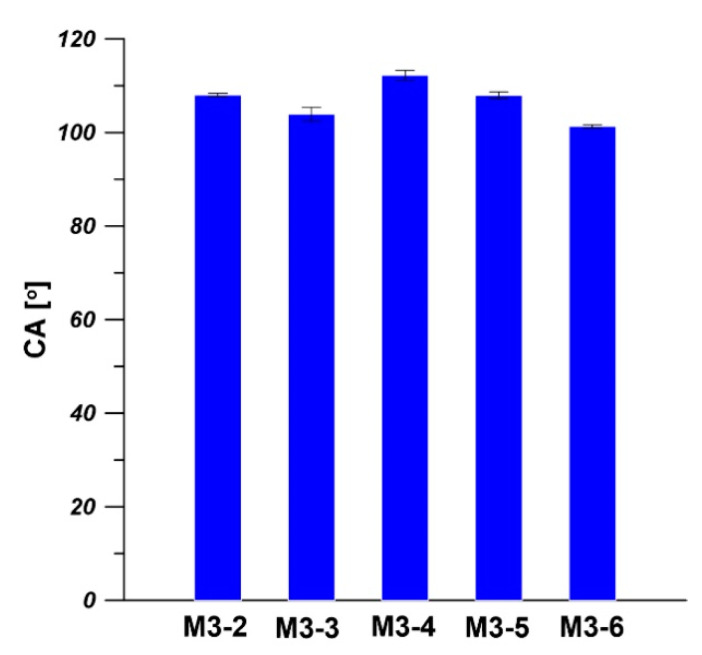
Water contact angle (CA) of the outer surface of the PDMS/PEI composite hollow fiber membrane. The dip coating conditions: PDMS solution concentration—15 wt% and coating time—10 min.

As is shown in Figure 13, the CAs of the outer surfaces of the prepared composite hollow fiber membranes are in the range of 103°–112° indicating the hydrophobicity of the outer surface. The CAs of the outer surface of hollow fiber substrates are between 84° and 89° (Figure 7 and Figure 10). The increase in CA is due to the formation of the elective PDMS layer on the outer surface of hollow fiber substrate. Our results are in agreement with the literature values of CA of PDMS membranes [51,52,53,54]. Knozowska et al. [51] and Kujawska et al. [52] found that the CA of flat sheet pristine PDMS membrane was 115°. While Khorasani et al. [53] and Lin et al. [54] found that the CAs of native PDMS membranes were 105° and 108°, respectively. It was reported that the wide range of CA values (95°–120°) for PDMS samples results from various experimental conditions, such as surface roughness and type of the substrate surface [52].

#### 3.2.2. Gas Separation Performance of the Composite Membranes

**Figure 14:**
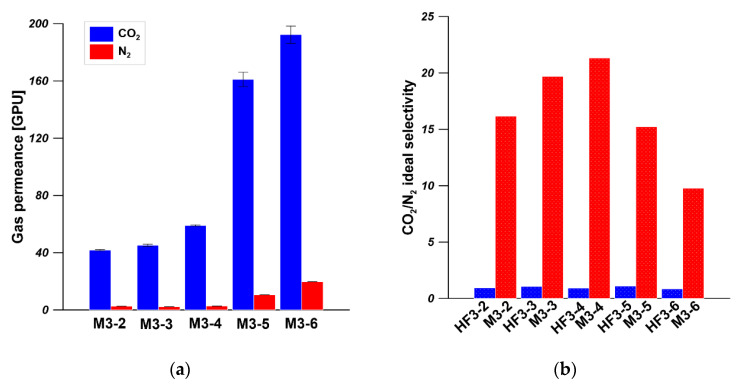
(**a**) The gas permeance of PDMS/PEI composite membranes. (**b**) The ideal selectivity of PEI hollow fibers and PDMS/PEI composite membranes. The dip coating conditions: PDMS solution concentration—15 wt% and coating time—10 min.

Figure 14 shows the CO_2_ and N_2_ permeances and CO_2_/N_2_ ideal selectivity of five types of PEI hollow fibers, spun from 20 wt% of PEI solution at various spinning conditions, coated with PDMS layer. As can be clearly seen, the CO_2_/N_2_ ideal selectivity of PEI hollow fibers was significantly enhanced after PDMS coating. The PDMS/PEI composite membranes M3-2, M3-3, M3-4, M3-5, and M3-6 possessed CO_2_/N_2_ ideal selectivity of 16, 20, 21, 15, and 10, respectively. The CO_2_ permeances of PDMS/PEI composite membranes M3-2, M3-3, M3-4, M3-5, and M3-6 were 41, 45, 59, 161, and 192 GPU, respectively. In comparison to the CO_2_ permeance of PEI hollow fibers in the range of 5000–8000 GPU, the CO_2_ permeance of PDMS/PEI composite membranes was significantly reduced. These findings indicated that the presence of a PDMS selective layer on the outer surface of PEI hollow fibers plays a crucial role in covering the defects or pores on the surface and forming a gas separation layer successfully. Consequently, the gas transport rate was reduced, and the CO_2_/N_2_ ideal selectivity was improved, indicating the trade-off relationship between permeance and selectivity [55]. By comparing the permeances of CO_2_ and N_2_, it was found that PDMS coated membranes possessed a higher affinity to CO_2_ rather than N_2_. Similar results were found in other research works [22,27]. The gas transport through the PDMS layer can be explained by using the solution–diffusion model. The high permeance of CO_2_ in the PDMS layer mainly resulted from the higher solubility coefficient in PDMS [23].

The reported CO_2_/N_2_ ideal selectivities of PDMS and PEI membranes are 9.5 and 23–35, respectively [56,57]. M3-4 possessed the CO_2_/N_2_ ideal selectivity of 21, which is much higher than the intrinsic selectivity of PDMS. This is because the PDMS coating efficiently sealed the non-selective pinholes (defects) on the skin layer of PEI hollow fibers [22,58]. The obtained CO_2_/N_2_ ideal selectivity of 21 is close to the reported CO_2_/N_2_ ideal selectivity of PEI membranes. Similar results were obtained in other research works [22,27,58]. Zulhairun et al. [58] dip coated a PDMS layer on the outer surface of polysulfone (PSF) hollow fibers to seal the pinholes and improve the gas separation performance. It was found that the CO_2_/N_2_ ideal selectivity was increased from 3.87 for pristine PSF hollow fiber to 31.34 for PDMS coated PSF hollow fiber. Madaeni et al. [27] found that the CO_2_/N_2_ ideal selectivity of PDMS coated polyethersulfone (PES) flat sheet membrane increased from 5.9 to 45.5 by increasing the number of coating layers from 2 to 5. This is because the repetition of coating resulted in good sealing of the defect holes and reduced the unselective gas transport.

PDMS coating is an effective way to prepare composite hollow fiber membranes with high gas separation performance. However, the use of different hollow fiber supports for the coating process might affect the gas separation of the composite hollow fiber membranes. In this study, different types of PEI hollow fiber spun at various spinning conditions were chosen as supports, and the PDMS layer was coated on the outer surface of PEI hollow fibers at the same coating conditions. As is shown in Figure 14, the CO_2_ permeances and CO_2_/N_2_ ideal selectivities of PDMS/PEI composite membranes M3-2, M3-3, and M3-4 slightly increased when the bore fluid flow rate increased from 6 mL/min to 12 mL/min, indicating that the bore fluid flow rate slightly affects the gas permeance of PDMS/PEI composite membranes, especially at higher bore fluid flow rates i.e., 9 and 12 mL/min. The slight increase in gas permeance with the increase in bore fluid flow rate might result from the weaker PDMS solution intrusion in the dip coating process. This is because the wall thickness of hollow fiber decreased (Figure 6), and the polymer chains became more packed when the bore fluid flow rate was higher. As a result, the PDMS solution intrusion was inhibited to some extent. In the comparison of the CO_2_ permeances and CO_2_/N_2_ ideal selectivities of PDMS/PEI composite membranes M3-3, M3-5, and M3-6, it was found that the addition of NMP into bore fluid significantly increased the CO_2_ permeances from 59 GPU to 192 GPU, while decreasing the CO_2_/N_2_ ideal selectivities from 21 to 10. This is attributed to the formation of a more porous inner surface of PEI hollow fiber (S3) and decreased skin layer on the inner and outer surface of PEI hollow fiber (Figure 9). On the other hand, the high PDMS concentration (15 wt%) resulted in less intrusion to the substructure of hollow fibers because of its high bulk viscosity [59]. The effect of the change of substructure and the formation of a more porous inner surface of hollow fiber predominantly resulted in the increase in CO_2_ permeances.

As was discussed above, it is concluded that the change of spinning parameters can manipulate the characteristics of structure and morphology of hollow fibers, resulting in significant influence on the CO_2_ permeance and selectivity of the prepared composite hollow fiber membranes. In this work, the hollow fibers spun at a higher bore fluid flow rate (12 cm^3^/min) and from bore fluid containing 50 wt% of NMP are desirable hollow fiber substrates for PDMS coating to produce good balance between gas permeance and ideal selectivity. Liang et al. [29] coated PDMS solution (0.3 wt%) on the outer surface of hollow fibers fabricated from various concentrations (17.5, 20.0, 22.5, and 25.0 wt%) of PAN solution and found that the CO_2_ permeance decreased from 5000 GPU to 1500 GPU with the increase in PAN concentration, while the CO_2_/N_2_ ideal selectivity was maintained around 10 [29]. Li et al. [23] investigated the effects of substrate characteristics on PAN–PDMS composite hollow fiber membranes for CO_2_/N_2_ and O_2_/N_2_ separation. It was found that when the PAN hollow fiber is made from solvent exchange post treatment, the selectivities of CO_2_/N_2_ and O_2_/N_2_ of the composite membrane are similar to the PDMS intrinsic selectivities, indicating the formation of a defect-free PDMS layer. When the PAN hollow fiber is made from freeze drying post treatment, the selectivities of CO_2_/N_2_ and O_2_/N_2_ of the composite membrane are much lower [23].

#### 3.2.3. The Comparison of Gas Separation Performance with Literature Data

As is shown in Table 4, the gas separation performances of the prepared PDMS/PEI composite membranes in this work are comparable with the gas separation performances of PDM- coated hollow fiber composite membranes in the literature, indicating the successful formation of a defect-free PDMS selective layer on PEI hollow fibers. The PDMS coated hollow fiber composite membranes either show higher gas permeance with lower selectivity or vice versa. The prepared PDMS/PEI composite membranes in this work followed the same trend since only a PDMS layer was coated on the outer surface of hollow fibers. To break the trade-off relationship between gas permeance and selectivity [55], some other strategies should be applied, for example, the incorporation of inorganic particles into the polymer matrix [60].

## 4. Conclusions

PEI hollow fibers were successfully fabricated by using a dry-jet-wet spinning technique. The polymer concentration significantly influenced the pore structure, skin layer thickness, and outer diameter of hollow fibers, which was confirmed by the significant decrease in gas permeance with the increase in polymer concentration. The bore fluid flow rate imparted predominant effects on the wall thickness and inner skin layer thickness rather than the outer diameter, gas permeance, and the structure of hollow fibers. The addition of NMP into bore fluid resulted in the decrease in the length of finger-like macrovoids near the lumen side due to the weakened nonsolvent intrusion and the decreased driving force of water inflow and solvent outflow. Consequently, the gas permeance of hollow fibers increased due to the formation of more porous inner surface and the decrease in skin layer thickness.

PDMS/PEI composite hollow fiber membranes were successfully prepared by dip-coating PDMS solution on the outer surface of hollow fibers spun from different spinning conditions. The gas separation performance of the composite membranes was affected by the hollow fiber substrates. Composite membranes M3-4 and M3-5 exhibited CO_2_ permeance of 59 GPU and 161 GPU, CO_2_/N_2_ selectivity of 21.3 and 16.2, respectively. The gas separation performance of M3-4 and M3-5 is comparable to the gas separation performance of PDMS coated hollow fiber membranes in the literature. The PDMS intrusion phenomenon occurred in the dip-coating process and was confirmed by elemental analysis.

## Figures and Tables

**Table 1:** The names of prepared hollow fibers and their corresponding fabrication parameters.

Hollow Fibers	PEI (wt%)	Bore Fluid	Flow Rate of Bore Fluid (cm^3^/min)
HF1	16	H_2_O	6
HF2	18	H_2_O	6
HF3-1	20	H_2_O	3
HF3-2	20	H_2_O	6
HF3-3	20	H_2_O	9
HF3-4	20	H_2_O	12
HF3-5	20	H_2_O/NMP * 50/50 (wt%)	9
HF3-6	20	H_2_O/NMP 30/70 (wt%)	9
HF4	22	H_2_O	6
HF5	24	H_2_O	6

* NMP—N-methyl-2-pyrrolidone.

**Table 2:** Spinning parameters for polyetherimide (PEI) hollow fiber fabrication.

Spinning Parameters	Spinning Conditions
Spinneret dimensions, OD/ID * (mm/mm)	4.8/2.1
Dry air gap length (cm)	25
Dope extrusion rate (mL/min)	7.6
Take up	Free fall
External coagulant	water
Temperature of external coagulant (°C)	25 ± 2
Temperature of spinneret (°C)	25 ± 2

* OD/ID—outer/inner diameter.

**Table 3:** The designations of polydimethylsiloxane (PDMS)/PEI composite membranes and hollow fiber substrates.

Hollow Fiber Substrate	PDMS/PEI Composite Membrane
HF3-2	M3-2
HF3-3	M3-3
HF3-4	M3-4
HF3-5	M3-5
HF3-6	M3-6

**Table 4:** The comparison of gas separation performance of the prepared composite membranes with the literature.

Membrane Materials	Permeance of CO_2_ (GPU)	CO_2_/N_2_ Selectivity	Pure Gas Permeance Testing Conditions	Ref.
PDMS/PAN	858	8.4	30 °C, 2 bar	[61]
	1473	8.1		
	1986	6.4		
PDMS/PAN	370	13.0	25 °C, 1 bar	[23]
PDMS/PAN	1926	10.4	25 °C, 2 bar	[62]
PDMS–Cu_3_(BTC)_2_/PSF	109	31.0	25 °C, 5 bar	[58]
PDMS/PSF	64	32	25 °C, 5 bar	[58]
PDMS/PSF	55	35.2	25 °C, 5 bar	[22]
PDMS/PSF	200	33.3	25 °C, 13.6 bar	[63]
PDMS/PES-PI	60	39	25 °C, 4 bar	[64]
PTFPMS/PEI	62	17.2	25 °C, 3 bar	[25]
PDMS/PEI	45	19.7	25 °C, 2 bar	This work
	59	21.3		
	161	16.2		

## Data Availability

Data is contained within the article or supplementary material.

## Supplementary Materials

The following are available online at https://www.mdpi.com/2077-0375/11/1/56/s1, Figure S1: SEM micrographs of outer and inner surfaces of PEI hollow fibers spun from different concentrations of polymer solution—A) HF1—16 wt%, (B) HF2—18 wt%, (C) HF3-2—20 wt%, (D) HF4—22 wt%, (E) HF5—24 wt%. Figure S2: SEM micrographs of outer and inner surface of PEI hollow fibers spun at different flow rates of bore fluid. (A) HF3-1—3 cm^3^/min, (B) HF3-2—6 cm^3^/min, (C) HF3-3—9 cm^3^/min, (D) HF3-4—12 cm^3^/min. Figure S3: SEM micrographs of outer and inner surfaces of PEI hollow fibers spun with different compositions of bore fluid. (A) HF3-3—H_2_O, (B) HF3-5—H_2_O/NMP 50/50 wt%, (C) HF3-6—H_2_O/NMP 30/70 wt%.
