# Supplementary material for: The Effects of PEI Hollow Fiber Substrate Characteristics on PDMS/PEI Hollow Fiber Membranes for CO2/N2 Separation
Source: Membranes (Basel). 2021 Jan 14;11(1):56. doi: 10.3390/membranes11010056 (PMC7828792; doi:10.3390/membranes11010056)
Supplement: Supplementary file 1 [file membranes-11-00056-s001.pdf]

Supporting Information

The Effects of PEI Hollow Fiber Substrate Characteristics on PDMS/PEI Hollow Fiber Membranes for CO_2/N_2 Separation

Guoqiang Li [1], Wojciech Kujawski [1,2,*], Katarzyna Knozowska [1] and Joanna Kujawa [1]

[1] Faculty of Chemistry, Nicolaus Copernicus University in Toruń, 7, Gagarina Street, 87-100 Toruń, Poland
[2] National Research Nuclear University MEPhI, 31, Kashira Hwy, 115409 Moscow, Russia
* Correspondence: kujawski@chem.umk.pl; Tel.: +48-566114517

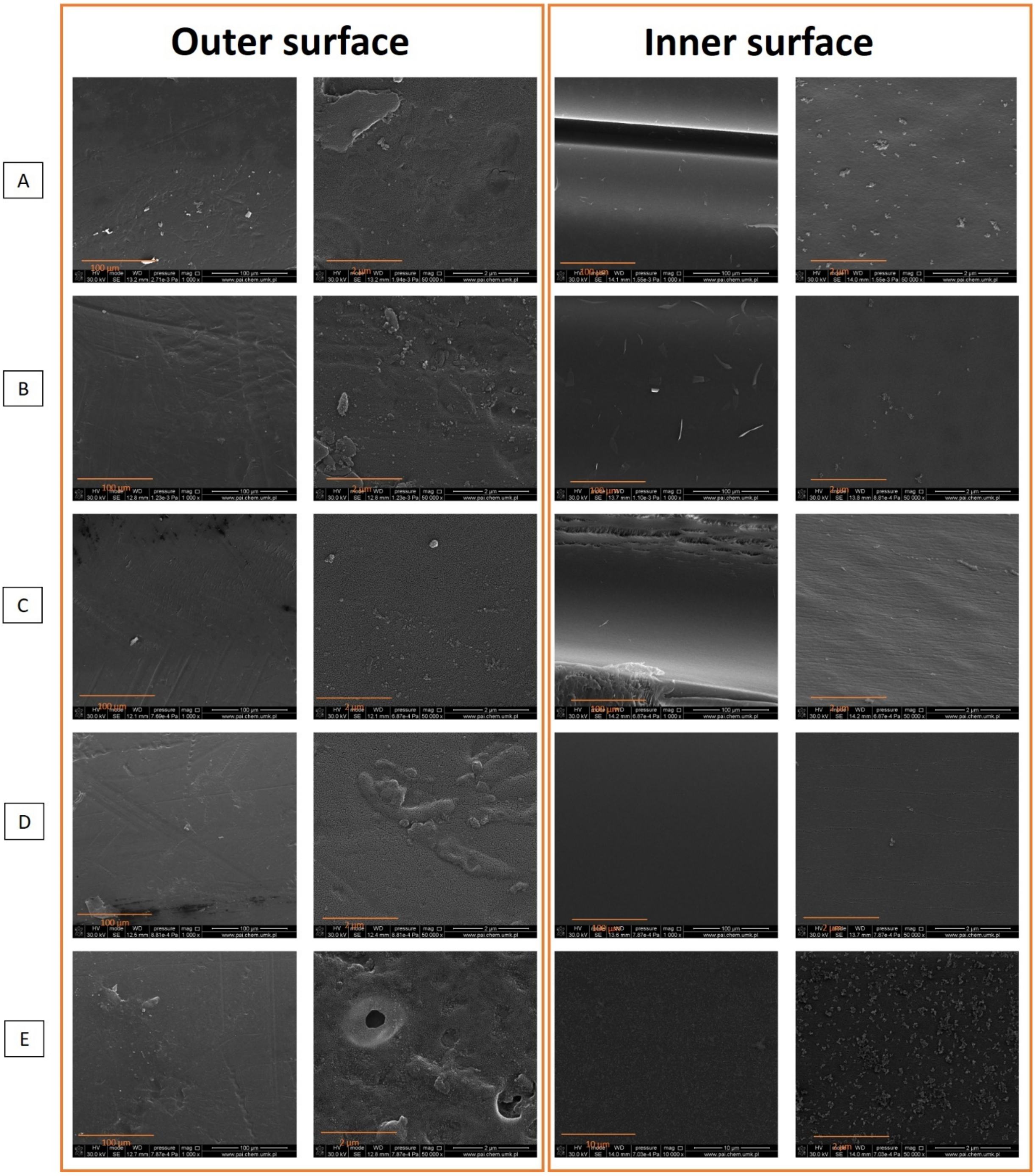

Figure S1. SEM micrographs of outer and inner surfaces of PEI hollow fibers spun from different concentrations of polymer solution—(**A**) HF1—16 wt.%, (**B**) HF2—18 wt.%, (**C**) HF3-2—20 wt.%, (**D**) HF4—22 wt.%, (**E**) HF5—24 wt.%.

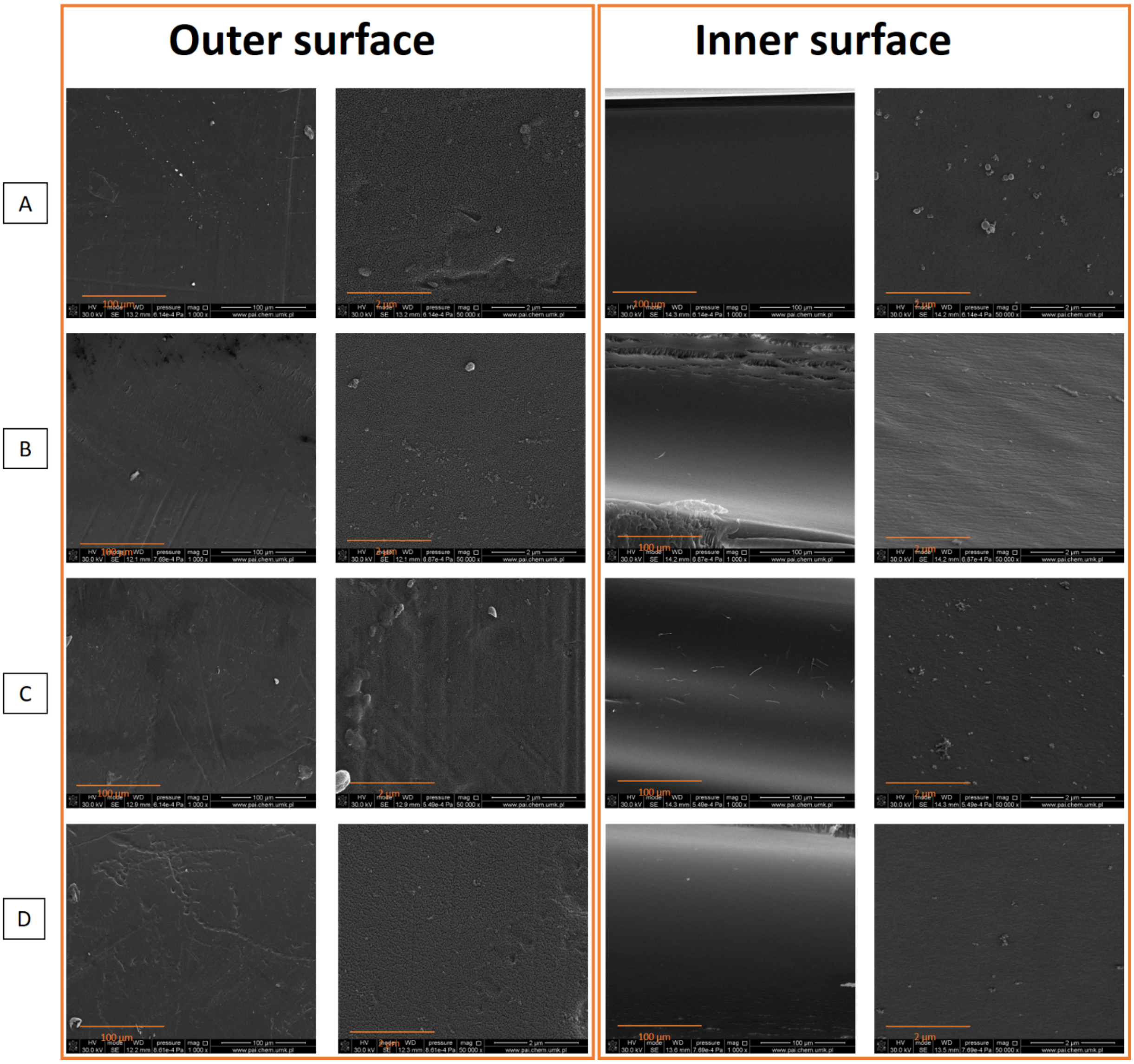

Figure S2. SEM micrographs of outer and inner surface of PEI hollow fibers spun at different flow rates of bore fluid. (A) HF3-1—3 cm^3/min, (B) HF3-2—6 cm^3/min, (C) HF3-3—9 cm^3/min, (D) HF3-4—12 cm^3/min.

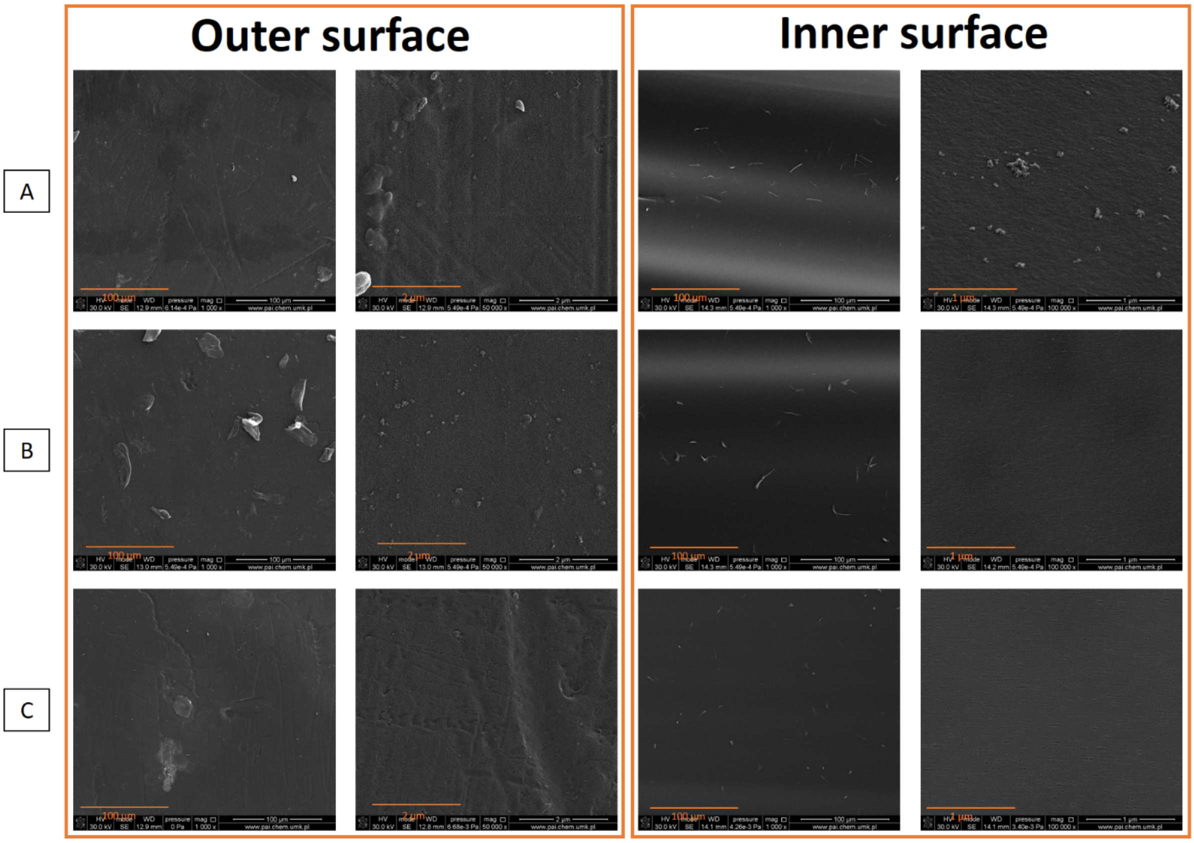

Figure S3. SEM micrographs of outer and inner surfaces of PEI hollow fibers spun with different compositions of bore fluid. (A) HF3-3—H_2O, (B) HF3-5—H_2O/NMP 50/50 wt.%, (C) HF3-6—H_2O/NMP 30/70 wt.%.
